# Supplementary figures and images for: Comparison of Metal-Based PZT and PMN–PT Energy Harvesters Fabricated by Aerosol Deposition Method
Source: Sensors (Basel). 2021 Jul 12;21(14):4747. doi: 10.3390/s21144747 (PMC8309660; doi:10.3390/s21144747)

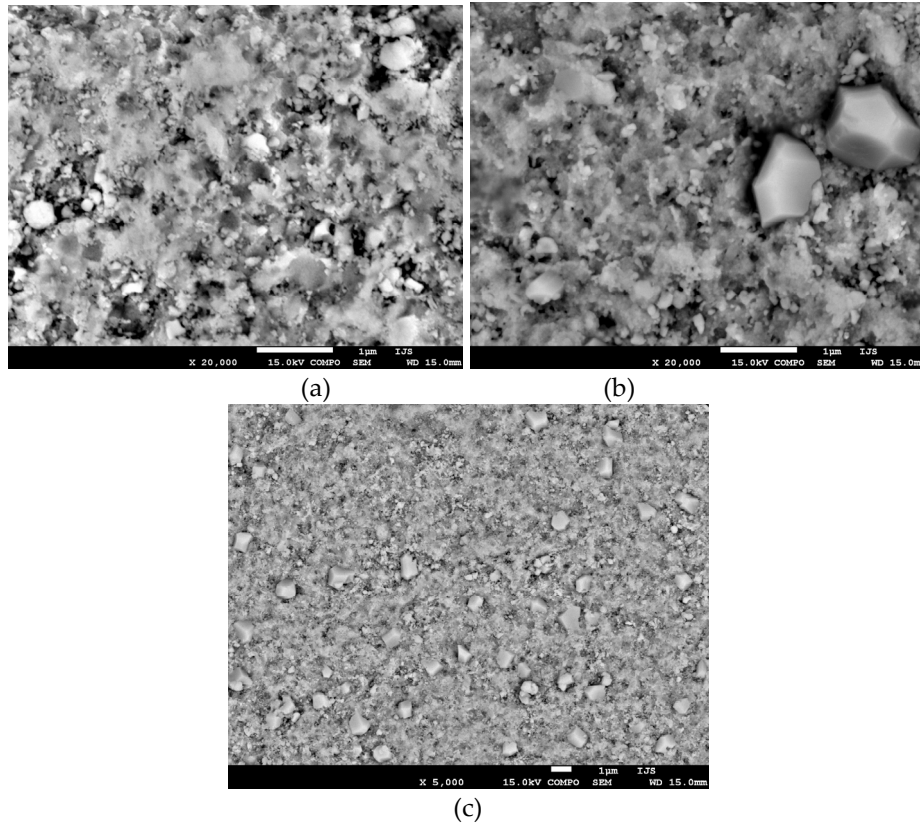

**Figure S1.** SEM images of the (a) 550 °C-annealed and (b) 600 °C-annealed, and (c) 650 °C-annealed PZT films.

Supplement: Supplementary file 1 [file sensors-21-04747-s001.zip › sensors-1279080-supplementary.pdf]
